# Microglial responses to CSF1 overexpression do not promote the expansion of other glial lineages

**DOI:** 10.1186/s12974-021-02212-0

**Published:** 2021-07-19

**Authors:** Ishani De, Vilena Maklakova, Suzanne Litscher, Michelle M. Boyd, Lucas C. Klemm, Ziyue Wang, Christina Kendziorski, Lara S. Collier

**Affiliations:** 1 Molecular and Cellular Pharmacology Graduate Program, University of Wisconsin, Madison, USA; 2 Pharmaceutical Sciences Division, School of Pharmacy, University of Wisconsin, Madison, USA; 3 Department of Statistics, University of Wisconsin, Madison, USA; 4 Department of BiostatisticsUniversity of Wisconsin, Madison, USA; 5 University of Wisconsin Carbone Comprehensive Cancer Center, Madison, USA

**Keywords:** CSF1, Microglia, Astrogliosis, Oligodendrogenesis

## Abstract

**Background:**

Colony-stimulating factor 1 (CSF1) expression in the central nervous system (CNS) increases in response to a variety of stimuli, and CSF1 is overexpressed in many CNS diseases. In young adult mice, we previously showed that CSF1 overexpression in the CNS caused the proliferation of IBA1^+^ microglia without promoting the expression of M2 polarization markers.

**Methods:**

Immunohistochemical and molecular analyses were performed to further examine the impact of CSF1 overexpression on glia in both young and aged mice.

**Results:**

As CSF1 overexpressing mice age, IBA1^+^ cell numbers are constrained by a decline in proliferation rate. Compared to controls, there were no differences in expression of the M2 markers ARG1 and MRC1 (CD206) in CSF1 overexpressing mice of any age, indicating that even prolonged exposure to increased CSF1 does not impact M2 polarization status in vivo. Moreover, RNA-sequencing confirmed the lack of increased expression of markers of M2 polarization in microglia exposed to CSF1 overexpression but did reveal changes in expression of other immune-related genes. Although treatment with inhibitors of the CSF1 receptor, CSF1R, has been shown to impact other glia, no increased expression of oligodendrocyte lineage or astrocyte markers was observed in CSF1 overexpressing mice.

**Conclusions:**

Our study indicates that microglia are the primary glial lineage impacted by CSF1 overexpression in the CNS and that microglia ultimately adapt to the presence of the CSF1 mitogenic signal.

**Supplementary Information:**

The online version contains supplementary material available at 10.1186/s12974-021-02212-0.

## Introduction

CNS resident macrophages (microglia) are known to have important roles in both CNS homeostasis and disease. Macrophage lineage cells including microglia express CSF1R, a receptor tyrosine kinase, which is activated by its ligands CSF1 and Interleukin-34 (IL-34). Mice genetically deficient for CSF1R have severe reductions in several macrophage populations including microglia [1]. In the normal brain, CSF1 and IL-34 have different expression patterns and therefore have regional-specific impacts on microglia [2–5]. During mouse neonatal development, whole-brain expression of *Csf1r* and its ligands peak during the 2nd and 3rd postnatal weeks, respectively, before declining [6]. This corresponds with the time that microglial numbers undergo rapid developmental changes. Specifically, microglial numbers peak during the 2nd postnatal week and then decline during the 3rd postnatal week due to both decreased proliferation and increased apoptosis [6]. In the adult, microglia do continue to proliferate at a low rate, but it has been observed that this proliferation is balanced by a similar rate of apoptosis [7]. Studies in mouse models utilizing CSF1R inhibitors indicate that the CSF1R signaling axis is important for both microglial proliferation and survival in the normal adult [7–9]. In situations of disease or injury, *Csf1* expression is often upregulated, which can influence microglial homeostasis [10–12].

Activated macrophages, including microglia, can be classified as being polarized to an M1 (pro-inflammatory) or M2 (immunosuppressive) phenotype [13]. In in vitro macrophage cultures, CSF1 has been proposed to promote M2-like phenotypes [14–16]. In the context of a high-grade brain tumor model where glioma-associated macrophages/microglia (GAMs) were M2 polarized, CSF1R inhibitors were found to decrease expression of M2 markers in GAMs such as *Arg1* and *Mrc1* (CD206) without influencing their numbers [17, 18]. However, the M1/M2 classification is highly simplified, and a wide variety of activation states for macrophages have been found [19, 20].

In normal adult mice, CSF1R expression is reported to be confined to microglia and some neurons [3, 12], yet treatment with CSF1R inhibitors can impact other glia. For example, increases in expression of astrocytic markers such as *Gfap* were observed upon treatment with a CSF1R inhibitor in some, but not all, studies [8, 21]. Decreased numbers of oligodendrocyte lineage cells were observed in certain brain regions in both *Csf1r* deficient mice and mice treated with certain CSF1R inhibitors [9, 22]. However, CSF1R inhibitor-mediated microglial depletion can be achieved without impacting oligodendrocyte lineage cells, suggesting potential off-target effects of these inhibitors [22]. Nevertheless, microglia have been shown to produce factors that influence oligodendrocyte precursor cell (OPC) proliferation, survival, or differentiation [23, 24]; however, it is not known if increasing microglial numbers is sufficient to impact oligodendrocyte lineage cells.

To study the role of increased CSF1 expression in the CNS, we previously generated transgenic mice that overexpress the secreted form of CSF1 in a subset of GFAP^+^ cells utilizing the TRE/tTA system (hereafter referred to as CSF1 OE mice). Previously, we examined the response of IBA1^+^ microglia to CSF1 OE in young adult mice [21]. Here, we expand upon those studies to examine responses to CSF1 OE in both microglia and other glia in young and aged mice.

## Materials and methods

### Mice

Mouse experiments were performed according to the institutional guidelines for animal care under the approval of the Institutional Animal Care and Use Committee of the University of Wisconsin, Madison. CSF1 OE mice have been described previously [21]. The genetic backgrounds of mice used for this study were F1s of CD1 to C57Bl/6 (immunofluorescence and CD11b^+^ cell enrichment) or C57Bl/6 (RNA isolation from half brain hemispheres).

### Fluorescence and immunofluorescence

For EGFP imaging, isolated brains were fixed in 4% PFA, sunk through sucrose, and embedded in OCT for frozen sectioning. Sections were washed in PBS before staining with DAPI for imaging. For immunofluorescence, slides from formalin-fixed, paraffin-embedded brains were rehydrated to water through a graded alcohol series and antigen retrieval performed in pH6 citrate buffer (Vector laboratories) with 0.02% TWEEN-20 added, following previously described procedures [21]. Antibodies and dilutions are described in Table 1. When needed, DyLight 649 labeled Lycopersicon Esculentum Lectin (DL-1178, Vector laboratories) was used at 1:300 before antibody staining. The Deadend TUNEL kit (Promega) was used to label apoptotic cells as previously described [21]. For cell counting in ImageJ [25, 26], images of z stacks of 10 steps 1 μ apart were used. For cell counting for each cell type, total cell numbers were determined by counting nuclei (DAPI). For cytoplasmic (IBA1 and GFAP) and cell surface (PDGFRA) antigens, a cell was considered positive if the signal surrounded the nucleus. For IBA1, GFAP, and OLIG2, PDGFA cell counts data are presented as the percent of total cells positive for the marker of interest (e.g., number of IBA1^+^ cells divided by the total number of cells times 100%). For IBA1 cell counts in the midbrain and brainstem, 10 60× fields were counted per brain region per mouse. For OLIG2, PDGFRA, or GFAP cell counting, a minimum of 1200 cells in the cerebellar white matter or cortex were counted. For IBA1^+^ cell proliferation, a minimum of 100 IBA1^+^ cells per brain region were examined per mouse. For microglial apoptosis, a minimum of 200 IBA1^+^ cells per brain region were examined per mouse.

**Table 1:** Antibodies and dilutions and utilized in this study

Primary antibodies						
Target	Host species	Catalog #	Manufacturer	Dilution	RRID	
IBA1	Rabbit	019-19741	Wako	1:200	AB_839504	
IBA1	Goat	ab48004	Abcam	1:150	AB_870576	
ARG1	Rabbit	ab91279	Abcam	1:200	AB_10674215	
MRC1 (CD206)	Rabbit	ab64693	Abcam	1:1000	AB_1523910	
Ki67	Mouse	550609	BD Biosciences	1:200	AB_393778	
OLIG2	Rabbit	AB9610	Millipore Sigma	1:150	AB_570666	
GFAP	Chicken	ab4674	Abcam	1:200	AB_304558	
GFAP	Rabbit	ab7260	Abcam	1:100	AB_305808	
CSF1	Goat	AF416	R&D systems	1:25	AB_355351	
PDGFRA	Goat	AF1062	R&D systems	1:200	AB_2236897	
Secondary antibodies						
Target	Host species	Catalog #	Manufacturer	Dilution	RRID	Conjugation
Anti-mouse	Goat	ab97239	Abcam	1:200	AB_10680851	FITC
Anti-rabbit	Donkey	ab150076	Abcam	1:200	AB_2782993	Alexa 594
Anti-chicken	Goat	ab150175	Abcam	1:200	AB_2732800	Alexa 647
Anti-goat	Donkey	ab150129	Abcam	1:250	AB_2687506	Alexa 488
Anti-goat	Donkey	A21447	Invitrogen	1:200	AB_141884	Alexa 647
Anti-mouse	Horse	MKB-2225	Vector Laboratories	1:250	AB_2336564	biotin^a^

^a^ Followed by 1 h incubation in Streptavidin-FITC (eBioscience, 11-4317-87) at 1:100 dilution

*RRID* Research Resource Identifier

### Statistics

With the exception of RNA-seq analysis, Prism (GraphPad) was used to perform statistical analyses and to produce graphs. All data were analyzed by unpaired, two-tailed *t* test with the exception of brainstem 6-month apoptotic cells which were analyzed by Wilcoxon signed-rank test. In all figures, error bars indicate standard deviation. Unless otherwise indicated, *n* = 3 to 4 mice per group.

### RNA isolation from half brain hemispheres

Tissue was homogenized in TRIzol and purified using the TRIzol Plus RNA Purification Kit (Thermo Fisher) including an on-column DNAse digestion. Post-isolation, the TURBO DNA free kit (Thermo Fisher) was used to eliminate any residual contaminating genomic DNA before further analysis.

### Reverse transcription, qualitative PCR (RT-qPCR)

cDNA was generated with the High-Capacity cDNA Reverse Transcription Kit (Thermo Fisher). Real-time PCR was completed using Step One Plus Real-Time PCR System and Power UP SYBR green (Applied Biosystems). Gene expression was normalized to *Tbp* and 2^−ΔCt^values were calculated. Primers sequences are provided in Table 2. Data in figures are presented as relative expression levels compared to control mice which are normalized to one.

**Table 2:** Primers utilized in this study

Gene	Forward primer 5′-3′	Reverse primer 5′-3′
*Arg1*	AGACATCGTGTACATTGGCTTGCG	CCCAGCTTGTCTACTTCAGTCATGGA
*C3*	ACAAGAACACCCTCATCATCTAC	GGCTGGATAAGTCCCACATT
*Csf1*	GGCATCATCCTAGTCTTGCTG	ACCTGTCTGTCCTCATCCT
*Gfap*	ACATGCAAGAGACAGAGGAGTGGT	AGTCGTTAGCTTCGTGCTTGGCTT
*Mog*	GCTTCTTCAGAGACCACTCTT	GATAGGCACAAGTGCGATGA
Mrc1 *	TATCTCTGTCATCCCTGTCTCT	CAAGTTGCCGTCTGAACTGA
*Olig2*	AGCGAGCACCTCAATCTAAT	GGGATGATCTAAGCTCTCGAA
*Pdgfr alpha*	GACGAGACCATCGAGGACAT	GCCTCGGGAACTTTCTCTCT
Slc1a2 *	AAAGAATCGCCCACCACAT	CCATGCTCCTCATTCTCACAG
Tbp *	TTCACCAATGACTCCTATGACC	CAAGTTTACAGCCAAGATTCACG

* indicates primers ordered pre-designed from IDT

### Microglia enrichment

CSF1 OE or control mice (ages p14 or p15, *n* = 4 per group) were perfused with PBS following a fatal dose of pentobarbital sodium. Brains were isolated and bisected sagittally. Left brain hemispheres were formalin-fixed for other studies and microglia were enriched from the right hemisphere by pull-down utilizing CD11b-conjugated magnetic beads (Miltenyi Biotech) using published methods with the Percoll (GE Healthcare) method for myelin removal [5]. Cell pellets were suspended in TRIzol (Thermo Fisher) and RNA was purified using the TRIzol Plus Purification kit including an on-column DNAse digestion step (Thermo Fisher).

### RNA sequencing (RNA-seq)

RNA quality and quantity were assayed with the RNA 6000 Pico Kit (Agilent) and Quant-iT RiboGreen RNA Assay Kit (Thermo Fisher). Libraries for RNA-seq were generated using the TruSeq RNA Library Prep Kit v2 (Illumina). 2X125 reads were obtained from one lane of the HiSeq 2500 system (Illumina).

### RNA-seq data analysis

Reads were mapped back to the genome using the short read aligner Bowtie v1.0.0 [6], followed by RSEM v1.2.7 [7] to estimate gene expression. Analyses were carried out in R [8], a publicly available statistical analysis environment. Specific software packages were obtained from Bioconductor [9] unless otherwise noted. EBSeq v1.14.0 [10] was used with default parameters to calculate the posterior probability of a gene being differentially expressed (DE). A gene was identified as being DE if its posterior probability exceeded 0.95 (which controls the overall False Discovery Rate (FDR) at 5%) and the posterior fold change (estimated from the empirical Bayes model) was less than 0.7 (or greater than 1.43 (1/0.7)).

Functional annotations were performed using the Database for Annotation, Visualization and Integrated Discovery (DAVID) [27, 28], and data presented are terms with Benjamini–Hochberg corrected *p* values < 0.05 to control the FDR at 5%.

## Results

### Proliferation rates of IBA1^+^ cells decline over time in CSF1 OE mice

**Fig. 1:**
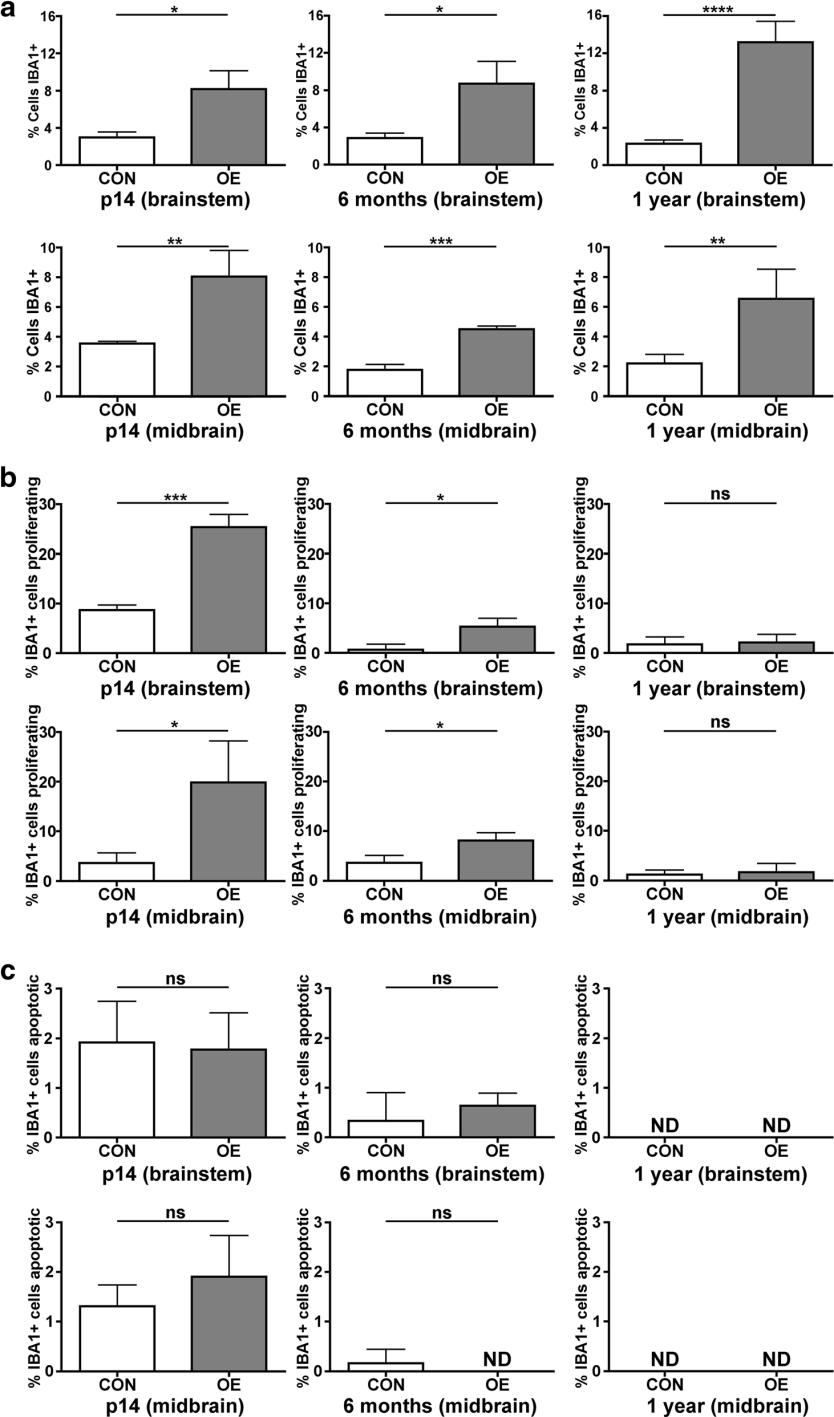
A decline in proliferation rate constrains IBA1^+^ cell numbers as CSF1 OE mice age. Cells were quantified in both the brainstem and midbrain for both control (CON, white bars) and CSF1 OE (OE, grey bars) mice. **A** Quantification of the percent of cells that are IBA1^+^. **B** Quantification of the percent of IBA1^+^ cells that are proliferating (Ki67^+^). **C** Quantification of the percent of IBA1^+^ cells that are apoptotic (TUNEL^+^ IBA1^+^). ND = none detected. ns = non-significant (*p* < 0.05); *p < 0.05; **p < 0.01; ***p < 0.001; and ****p < 0.0001 by unpaired, two-tailed *t* test with the exception of brainstem 6-month apoptotic cells which was analyzed by Wilcoxon signed-rank test

Previously, an increased rate of IBA1^+^ microglial proliferation and increased IBA1^+^ microglial numbers were observed in young adult CSF1 OE mice compared to controls [21]. Continued expansion of IBA1^+^ cells could have detrimental effects in the CNS; therefore, it was hypothesized that responses to CSF1 overexpression would need to change over time. To examine if adaptation to increased CSF1 levels occurs over time, IBA1^+^ cell counts and proliferation rates were examined in young and aged CSF1 OE and control mice (representative images in Supplemental Figure 1). To be consistent with a past study of CSF1 OE mice, IBA1 counts were performed in the brain stem and midbrain, two regions known to harbor high levels of CSF1 transgene expression [21]. IBA1 counts confirmed that IBA1^+^ cell numbers were increased in CSF1 OE mice compared to controls at all ages examined (Fig. 1A). Next, Ki67 staining was used to identify proliferating IBA1^+^ cells. At both p14 and 6 months, microglial proliferation rates were higher in CSF1 OE mice compared to controls, but by 1 year of age, microglial proliferation rates were equivalent (Fig. 1B). This difference in aged mice is not due to transgene silencing as RT-qPCR detects approximately 2.5-fold increased *Csf1* expression in p14 CSF1 OE mice compared to control, and an approximately 3-fold increase in *Csf1* expression in 1-year-old CSF1 OE mice compared to 1-year-old control mice (Supplemental Figure 2A and 2B). Additionally, EGFP (encoded as part of the TRE-CSF1 transgene) and CSF1 proteins are also readily detected in 1-year-old CSF1 OE mice (Supplemental Figure 2C and 2D). TUNEL analysis indicates that CSF1 OE does not influence apoptosis rates (Fig. 1C; representative images in Supplemental Figure 3), indicating that downregulation of the proliferative response likely constrains IBA1^+^ cell expansion in CSF1 OE mice.

### CSF1 OE does not impact the expression of the M2 polarization markers ARG1 and MRC1, even in aged mice

**Fig. 2:**
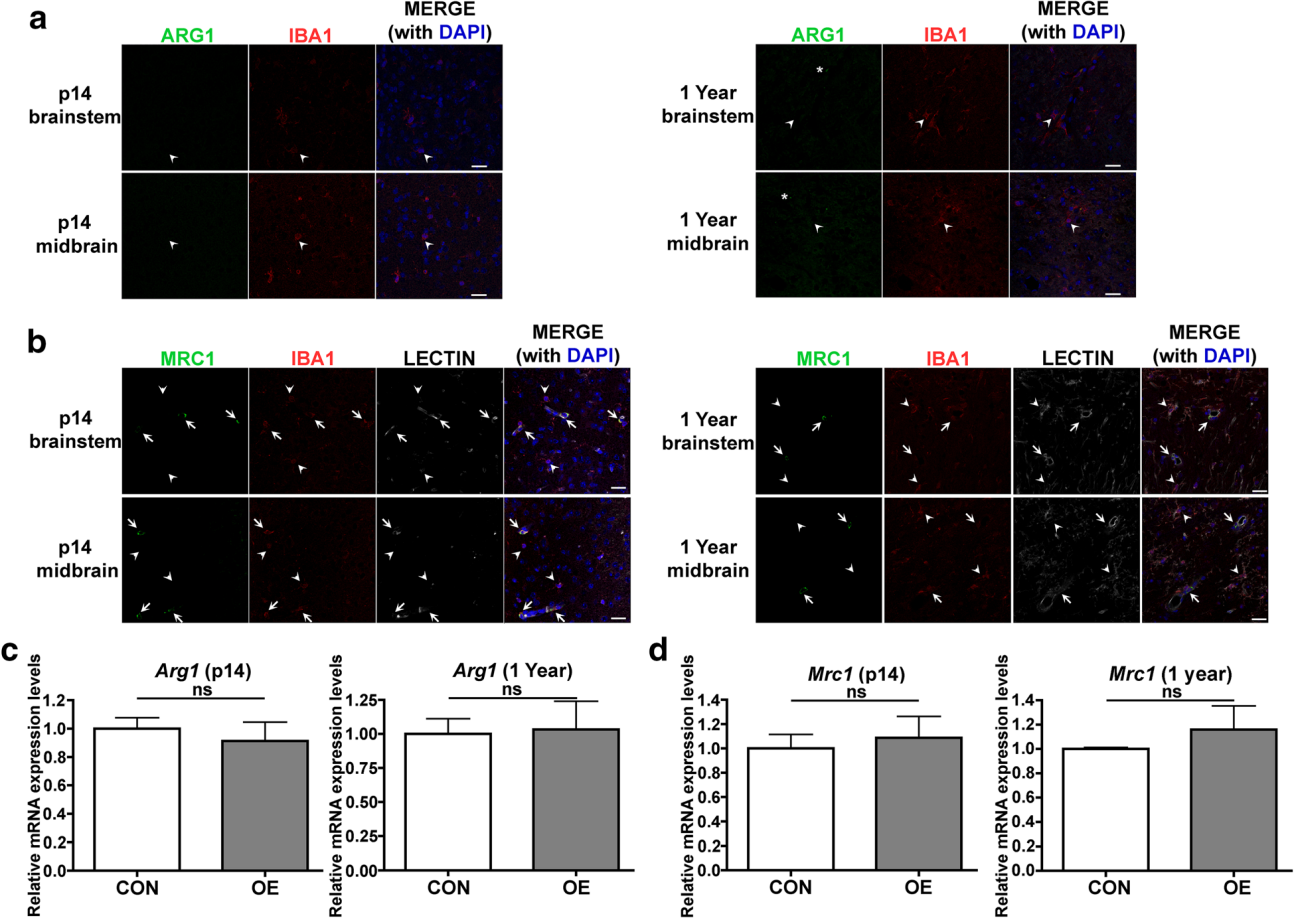
CSF1 OE does not increase expression of M2 markers, even in aged mice. **A** Representative images of immunofluorescence for ARG1 (green) with IBA1 (red) in the indicated brain regions in CSF1 OE mice at p14 and 1 year. DAPI (blue) is also shown in the merged image. Arrowheads indicate examples of IBA1^+^ ARG1^−^ cells while an asterisk indicates an example of lipofuscin autofluorescence that was also visible in additional channels (not shown). **B** Representative images of immunofluorescence for MRC1 (CD206) (green) with IBA1 (red) in the indicated brain regions in CSF1 OE mice at p14 and 1 year. Arrowheads indicate examples of IBA1^+^ MRC1^−^ parenchymal microglia and arrows indicate MRC1^+^ perivascular macrophages residing next to blood vessels that stain with lectin (in white, lectin staining is also present as expected in microglia). DAPI (blue) is also shown in the merged image. Scale bars = 25 μ. RT-qPCR for *Arg1* (**C**) and *Mrc1* (**D**) in p14 and 1-year mice on mRNA isolated from half brain hemispheres of control (CON, white shaded bars) or CSF1 OE (OE, grey shaded bars) mice. ns = non-significant (*p* > 0.05), unpaired, two-tailed *t* test

Although CSF1 has been proposed to be a factor that can polarize macrophages toward an M2 phenotype [14–16], increased expression of markers of M2 polarization was not previously observed in young adult CSF1 OE mice [21]. To determine if continued exposure to CSF1 OE promotes an M2 phenotype, immunofluorescence staining was performed for two commonly used M2 markers, ARG1 and MRC1 (CD206) [13], in young and aged mice. ARG1 was not detectable in IBA1^+^ cells in either p14 or 1-year OE mice (Fig. 2A; validation of ARG1 antibody efficacy can be found in Supplemental Figure 4). MRC1 expression as detected by immunofluorescence was observed, as expected, in perivascular macrophages but was not observed in parenchymal IBA1^+^ cells in either p14 or 1-year OE mice (Fig. 2B). RT-qPCR also did not detect a difference in *Arg1* (Fig. 2C) or *Mrc1* (Fig. 2D) expression between the brains of control and CSF1 OE mice at either p14 or 1 year. Taken together, this data provides additional evidence that CSF1 as the only stimulus does not impact microglial polarization toward an M2 phenotype in vivo.

### CSF1 OE impacts the expression of genes involved in translation and the immune response in microglia

**Fig. 3:**
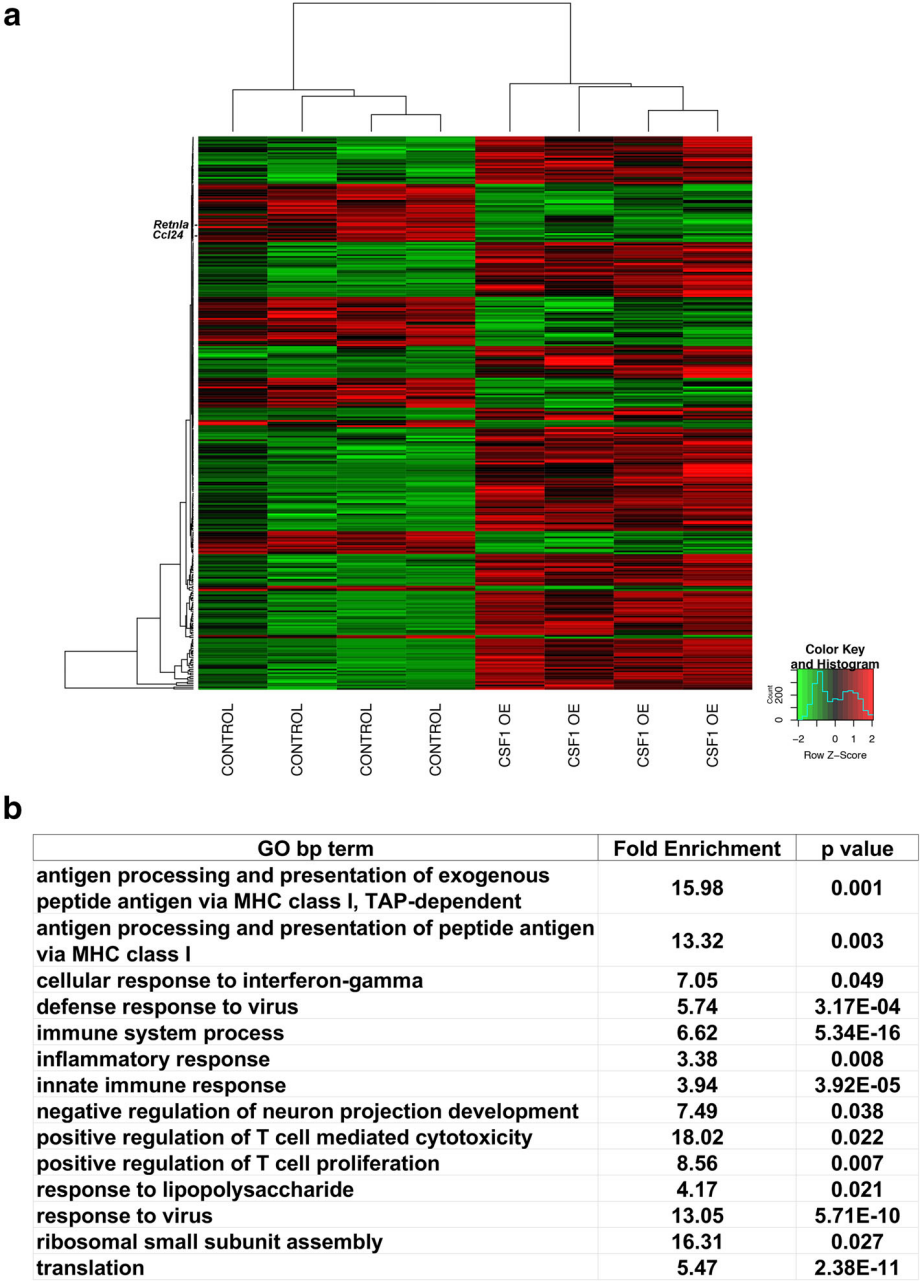
**A** Heatmap of 314 differentially expressed genes for control versus CSF1 OE samples. Each row represents a single gene, while each column represents a sample. Two hundred fourteen genes are upregulated (shown in red) in the CSF1 OE condition and one hundred are downregulated (shown in green). Genes are shown in the order depicted in Supplemental Table 1, and the rows for the M2 markers *Ccl24* and *Retnla* are indicated. **B** GO terms enriched in differentially expressed genes between microglia from CSF1 OE and control mice. Benjamini–Hochberg corrected *p* values are presented

To further examine the impact of CSF1 OE on microglia, CD11b-conjugated magnetic beads were used to enrich for microglia in CSF1 OE or control mice for RNA-seq. Three hundred fourteen genes were found to be differentially expressed between control and CSF1 OE (Fig. 3A and Supplemental Table 1). Hierarchical clustering found that samples of the same genotype grouped together. No commonly used markers for M2 phenotypes [29, 30] had increased expression in CSF1 OE microglia, and two (*Ccl24* and *Retnla* (*Fizz1*)) had decreased expression compared to control. To determine which cellular processes are impacted when microglia are exposed to CSF1 overexpression, functional annotation was performed using DAVID (Fig. 3B). Most GO biological process (bp) terms enriched in differentially expressed genes were related to either translation or immune system activities. Transcripts encoding multiple cytoplasmic ribosomal proteins were upregulated in CSF1 OE while several additional subunits had increased expression in CSF1 OE microglia but did not meet the threshold to be considered differentially expressed (data not shown). Other genes involved in ribosome biogenesis or translation, including *Eef1b2*, *Rbm3*, *Nhp2*, and *Npm1*, were also upregulated in CSF1 OE microglia. Multiple genes involved in different aspects of immune responses were also differentially expressed, including both transcripts encoding secreted molecules like Cfb and cell surface receptors like TLR2. *MHC class I* genes and additional interferon-regulated genes such as *Oas* family members and *Usp18* were also upregulated in microglia from CSF1 OE mice. In summary, RNA-seq data support the lack of increased expression of M2 polarization markers in microglia exposed to CSF1 OE; and also indicate that CSF1 OE exposed microglia do have some phenotypic differences from control microglia.

### CSF1 OE does not increase the expression of oligodendrocyte lineage markers

**Fig. 4:**
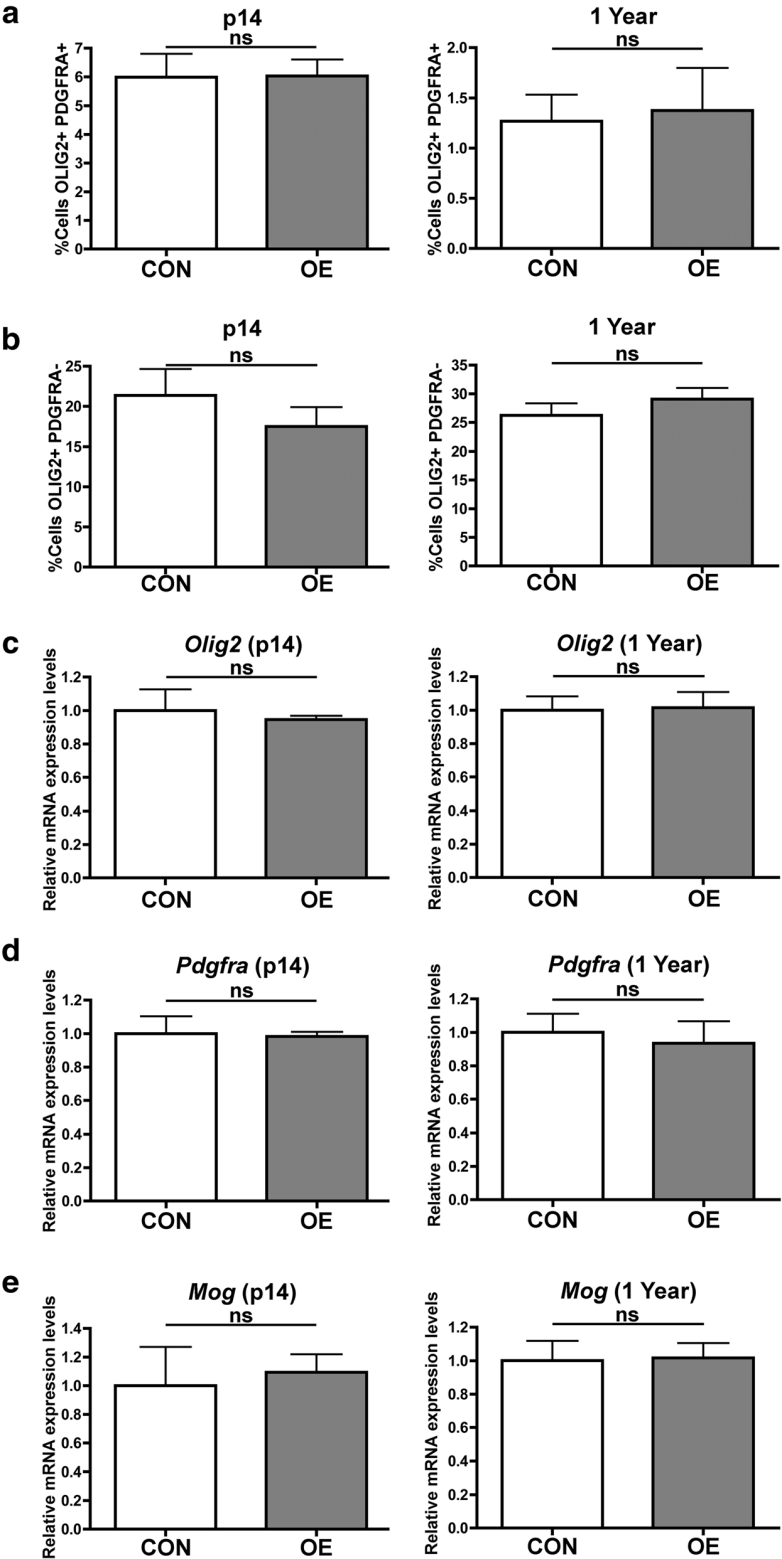
CSF1 OE does not impact the expression of genes expressed in the oligodendrocyte lineage. Quantification of the percent of cells that are OPCs (OLIG2^+^; PDGFRA^+^) (**A**) or mature or maturing oligodendrocytes (OLIG2^+^; PDGFRA^−^) (**B**) in the cerebellar white matter of control (CON, white shaded bars) or CSF1 OE (OE, grey shaded bars) at p14 and 1 year. RT-qPCR for *Pdgfra* (**C**), *Olig2* (**D**), and *Mog* (**E**) on mRNA isolated from half brain hemispheres of control (CON, white shaded bars) or CSF1 OE (OE, grey shaded bars) mice at p14 and 1 year. ns = non-significant (*p* > 0.05), unpaired, two-tailed *t* test

There are several lines of evidence indicating that normal microglia influence oligodendrogenesis [23, 24]. To determine if CSF1 overexpression impacts oligodendrocyte lineage cells, OLIG2^+^; PDGFRA^+^ oligodendrocyte precursor cells (OPCs) as well as maturing or mature oligodendrocyte lineage (OLIG2^+^; PDGFRA^−^) cells were quantified in the cerebellar white matter of CSF1 OE and control mice. This region was chosen because OLIG2^+^ cells were robustly depleted there in neonatal mice treated with a CSF1R inhibitor [9], and increased numbers of microglia were also observed in this brain region in both p14 and 1-year CSF1 OE mice compared to controls (Supplemental Figure 5). There were no differences in OLIG2^+^; PDGFRA^+^ or OLIG2^+^; PDGFRA^−^ cells in CSF1 OE mice compared to controls at either age (Fig. 4A, B; representative images in Supplemental Figure 6), and the proliferation rates of these cell types were also not statistically different between groups (Supplemental Figure 7). Similar results were found in the cortex (Supplemental Figure 8). Furthermore, RT-qPCR for markers of both oligodendrocyte precursor cells (*Pdgfra* and *Olig2*) as well as mature oligodendrocytes (*Mog*) did not detect differences between CSF1 OE and control mice (Fig. 4C) at either p14 or 1 year. Therefore, CSF1 OE and the resulting increase in IBA1^+^ cells do not appear to impact oligodendrocyte lineage cells.

### CSF1 OE does not impact the expression of astrocyte markers

**Fig. 5:**
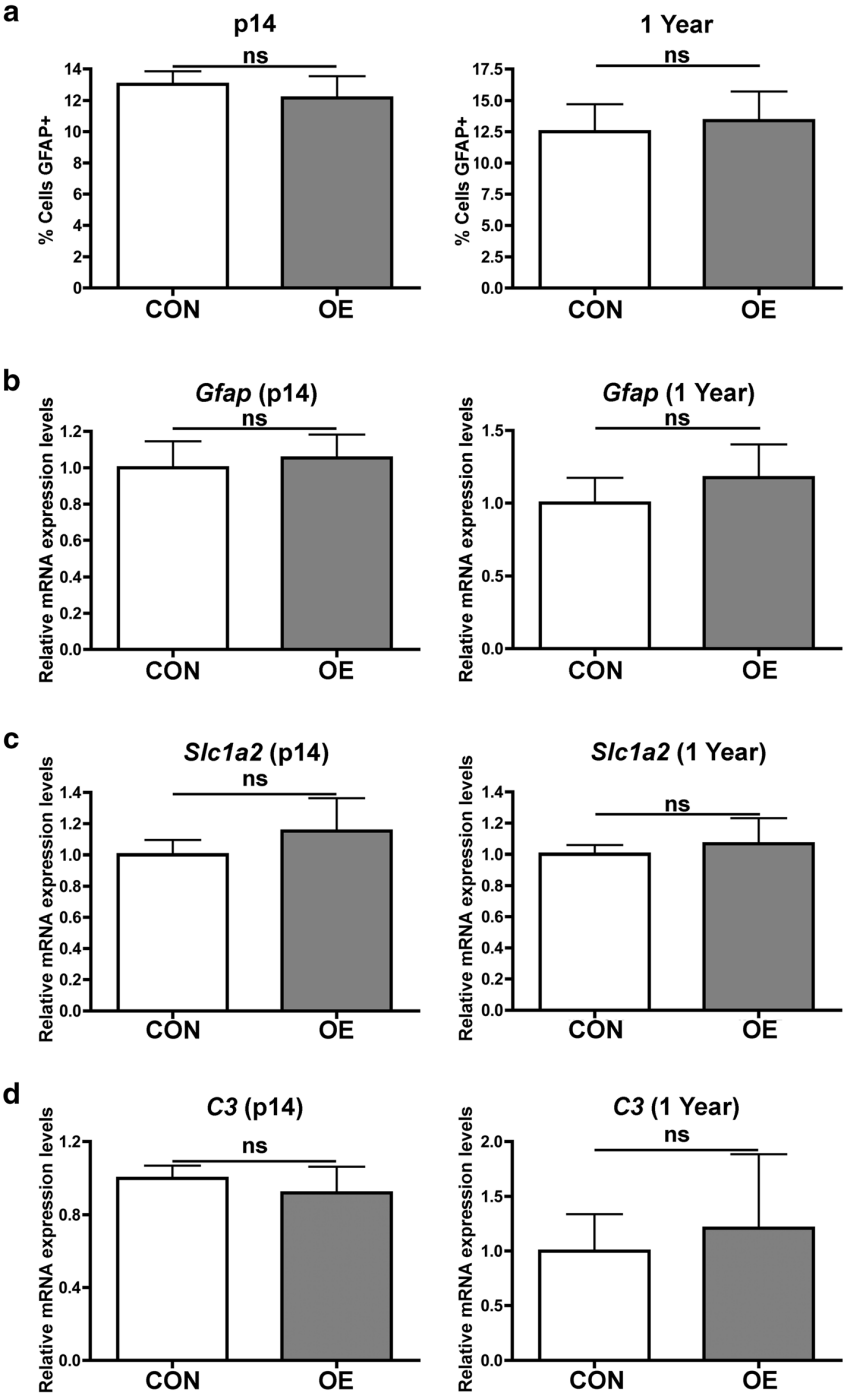
CSF1 OE does not impact the expression of astrocytic genes. **A** Quantification of the percent of cells that are GFAP^+^ astrocytes in the cerebellar white matter of control (CON, white shaded bars) or CSF1 OE (OE, grey shaded bars) at p14 and 1 year. RT-qPCR for *Gfap* (**B**), *Slc1a2* (**C**), and *C3* (**D**) on mRNA isolated from half brain hemispheres of control (CON, white shaded bars) or CSF1 OE (OE, grey shaded bars) mice at age p14 or 1 year. ns = non-significant (*p* > 0.05), unpaired, two-tailed *t* test

Some studies of CSF1R inhibitors have observed increased expression of astrocytic markers such as *Gfap* in response to the drug [8], and activated microglia have been shown to induce the formation of “A1”-activated astrocytes [31]. To determine if increasing CSF1 levels and microglia would also impact GFAP^+^ astrocyte numbers, GFAP^+^ cells were also quantified in the cerebellar white matter of CSF1 OE and control mice. No differences were observed in the percentage of cells that are GFAP^+^ in between the two groups at both p14 and 1 year (Fig. 5A; representative images in Supplemental Figure 9). Proliferating (Ki67^+^) GFAP^+^ astrocytes were very rare in p14 mice and not detected in 1-year-old mice (data not shown). Furthermore, no differences in expression of *Gfap* or *Slc1a2* (also known as *Glt1*, a glutamate transporter with enriched expression in astrocytes) were detected by RT-qPCR between CSF1 OE and control mice at either p14 or 1 year (Fig. 5B, C). Additionally, CSF1 OE did not impact expression levels of the “A1” astrocyte marker *C3* at either p14 or 1 year of age (Fig. 5D). Therefore, CSF1 OE and the resulting increase in IBA1^+^ cells do not appear to promote GFAP^+^ astrocyte expansion or activation.

## Discussion

Normal adult microglial numbers have been found to be maintained by equivalent apoptotic and proliferative rates. Blocking apoptosis does increase microglial numbers, but numbers eventually stabilize [7]. Our observation that IBA1^+^ cell proliferation rates decline over time in CSF1 OE mice indicates that a similar phenomenon occurs in the presence of a pro-proliferative stimulus. It is possible that there are mechanisms in place by which the brain is capable of sensing and responding to abnormal microglial density. It is also possible that CSF1-induced proliferation eventually leads to microglial senescence [32], or that older microglia respond differently to the CSF1 mitogenic signal. Additional studies will be required to distinguish between these possibilities.

In a murine glioma model where *Csf1* expression is increased approximately 2.5-fold compared to normal brain, CSF1R inhibitors decrease expression of M2 markers including *Arg1* in GAMs [17, 18], suggesting that increased CSF1/CSF1R signaling can promote polarization toward a M2 phenotype in the diseased CNS. However, in CSF1 OE mice we do not find evidence for increased expression of the commonly used M2 polarization markers ARG1 and MRC1 (CD206), even in aged mice. Gliomas produce other factors that signal to macrophage lineage cells, so one possible explanation is that increased levels of CSF1 alone are unable to increase expression of M2 polarization genes but can do so when combined with other signals.

RNA-seq data indicates that increased CSF1 signaling influences transcription of a relatively limited number of genes in microglia, several of which are related to protein synthesis. In bone marrow-derived macrophages in vitro, CSF1 has been shown to promote protein synthesis [12] and proliferating cells require increased protein synthesis. Additionally, “cellular response to interferon gamma (IFN-γ)” is one of the GO terms enriched in microglia from CSF1 OE mice. Given that IFN-γ is one of the stimuli used to polarize to a M1 phenotype [13], CSF1 OE microglia could therefore be considered to have some M1 characteristics. However, RNA-seq data indicate that the commonly used M1 marker *Nos2* (iNOS) is not differentially expressed in CSF1 OE microglia compared to control. Therefore, our data indicate that in vivo, CSF1 OE promotes a gene expression state in microglia that falls on the continuum between M1 and M2. Moreover, it is possible that CSF1 signaling is responsible for some of the previously recognized expression of interferon targets that occurs in microglia in normal mice [33]. Further studies will be necessary to fully elucidate how CSF1 OE influences immune responses in the CNS.

Microglial actions are known to have impacts on the oligodendrocyte lineage. For example, microglial specific deletion of *transglutaminase 2* decreased OPC proliferation and caused a reduction in OPC and oligodendrocyte numbers in otherwise wild-type mice [23]. Our studies indicate that increasing IBA1^+^ cells does not have the converse effect. It is possible that in the normal brain, microglial actions supporting oligodendrogenesis are already “saturated” and that further increasing their number has no impact. Alternatively, CSF1 OE may produce a state in IBA1^+^ cells that renders them incapable of providing oligodendrocyte support.

Similarly, in CNS injury or disease, both increased microglial numbers and an astroglial reaction are commonly observed. We utilized the marker GFAP to examine if CSF1 OE impacts astrocyte numbers. One limitation to our study is that some astrocytes, particularly those in the grey matter, do not express levels of GFAP that are detected by immunohistochemistry [34] and therefore would not have been detected by our methods. Upon activation, astrocytes can take on different phenotypes, and one such phenotype termed “A1” is induced by interleukin 1α (Il-1α); tumor necrosis factor (TNF); and complement component 1, subcomponent q (C1q) produced by activated microglia [31]. Our RNA-seq data did not find increased expression of transcripts encoding these factors in microglia and by RT-qPCR, we did not observe increased expression of the “A1” marker *C3*. Our data, therefore, support the hypothesis that altered microglial function, and not simply increased microglial density, contributes to the astrogliosis that occurs in CNS pathologies.

## Conclusions

In summary, our studies found no impact of CSF1 overexpression alone on glia outside of microglia. However, in situations of CNS disease or injury where multiple inflammatory mediators are produced, CSF1 overexpression could act together with other factors to have additional impacts.

## Supplementary Information

**Additional file 1: Supplemental Figure 1.** Representative images for IBA1 and Ki67 immunofluorescence. Genotype, age, and brain region are indicated for each image. Scale bar = 50 microns.

**Additional file 2: Supplemental Figure 2.** Transgene expression in CSF1 OE mice. RT-qPCR indicates increased *Csf1* levels in CSF1 OE (grey bars) mice compared to control mice (white bars) at both p14 (A) and 1 year (B). No-RT reactions were included for all samples and no amplification was detected (not shown). N=3-5 mice per group. ***= p<0.001; ****= p<0.0001; unpaired, two-tailed t-test. (C) Representative images showing expression of EGFP (green) in 1 year CSF1 OE mice. Scale bar = 50 microns. (D) Representative images showing detection of CSF1 protein by immunofluorescence in a subset of GFAP^+^ cells in 1-year old CSF1 OE mice but not control mice. Asterisks= examples of autofluorescence of red blood cells; arrows= examples of CSF1^+^ GFAP^+^ cells. Scale bar = 20 microns.

**Additional file 3: Supplemental Figure 3.** Representative images for IBA1 and TUNEL immunofluorescence. Genotype, age, and brain region are indicated for each image. Scale bar = 50 microns.

**Additional file 4: Supplemental Figure 4.** ARG1 antibody validation. ARG1 (green) and IBA1 (red) immunofluorescence staining in a murine glioma. Arrow indicates an example ARG1^+^ IBA1^+^ cell. Scale bar = 25 microns.

**Additional file 5: Supplemental Figure 5.** IBA1^+^ cell numbers are increased in the cerebellar white matter of CSF1 OE mice. Quantification of the percent of cells that are IBA1^+^ in (CON, white bars) and CSF1 OE (OE, grey bars) mice at p14 (A) and 1 year (B). **=*p*<0.01

**Additional file 6: Supplemental Figure 6.** Representative images for OLIG2, PDGFRA, and Ki67 immunofluorescence. Genotype and age are indicated for each image while dots indicate the edge of cerebellar white matter. Scale bar = 50 microns.

**Additional file 7: Supplemental Figure 7.** Proliferation rates of oligodendrocyte lineage cells in the cerebellar white matter do not differ between control (CON, white bars) and CSF1 OE (OE, grey bars) mice. Quantification of the percent of OPCs (PDGFRA^+^; OLIG2^+^) (A) or mature or maturing oligodendrocytes (OLIG2^+^; PDGFRA^-^) (B) cells that are proliferating (Ki67^+^) at p14. No proliferating oligodendrocyte lineage cells were observed in 1-year old mice of either genotype. ns= non-significant (*p*>0.05), unpaired, two-tailed t-test.

**Additional file 8: Supplemental Figure 8.** IBA1^+^ cells are increased but there are no differences in oligodendrocyte lineage cells in the cortex of CSF1 OE mice. Quantification of the percent of cells that are IBA1^+^ (A), OLIG2^+^; PDGFRA^+^ (OPCs) (B), and mature or maturing oligodendrocytes (OLIG2^+^; PDGFRA^-^) (C) cells in control (CON, white shaded bars), and CSF1 OE (OE, grey shaded bars) mice at p14 and 1 year. D) Quantification of the percent of OPCs (PDGFRA^+^; OLIG2^+^) or mature or maturing oligodendrocytes (OLIG2^+^; PDGFRA^-^) cells that are proliferating (Ki67^+^) in control (CON, white shaded bars) and CSF1 OE (OE, grey shaded bars) mice at p14. No proliferating oligodendrocyte lineage cells were observed in 1-year old mice of either genotype. ns= non-significant (*p*>0.05), *=*p*<0.05, **=*p*<0.01, unpaired, two-tailed t-test.

**Additional file 9: Supplemental Figure 9.** Representative images for GFAP and Ki67 immunofluorescence. Genotype and age are indicated for each image while dots indicate the edge of cerebellar white matter. Scale bar = 50 microns.

**Additional file 10: Supplemental Table 1.** Genes that were found to be differentially expressed in microglia from CSF1 OE mice compared to control (CON). PPDE= posterior probability of differential expression, FC= fold change. Normalized expected counts are shown for each gene for each of four samples from the two genotypes.

## Data Availability

RNA-sequencing data has been deposited at GEO (accession number GSE151698). Other data from this manuscript is available from the corresponding author upon reasonable request.

## Abbreviations

ARG1: Arginase 1
C3: Complement C3
CON: Control
CNS: Central nervous system
CSF1: Colony-stimulating factor 1
CSF1R: Colony-stimulating factor 1 receptor
DE: Differentially expressed
FDR: False discovery rate
FC: Fold change
GAM: Glioma-associated macrophages/microglia
IBA1: Ionized calcium-binding adaptor molecule 1
IFN-γ: Interferon gamma
IL-34: Interleukin -34
MRC1: Mannose receptor C-type 1
Ns: Not significant
ND: Not detected
OE: Overexpressing
OLIG2: Oligodendrocyte transcription factor 2
PDGFRA: Platelet-derived growth factor receptor A
PPDE: Posterior probability of differential expression
RT-qPCR: Reverse transcription, qualitative PCR
Seq: Sequencing
TNF: Tumor necrosis factor
