# Supplementary figures and images for: Microglial responses to CSF1 overexpression do not promote the expansion of other glial lineages
Source: J Neuroinflammation. 2021 Jul 19;18:162. doi: 10.1186/s12974-021-02212-0 (PMC8290555; doi:10.1186/s12974-021-02212-0)

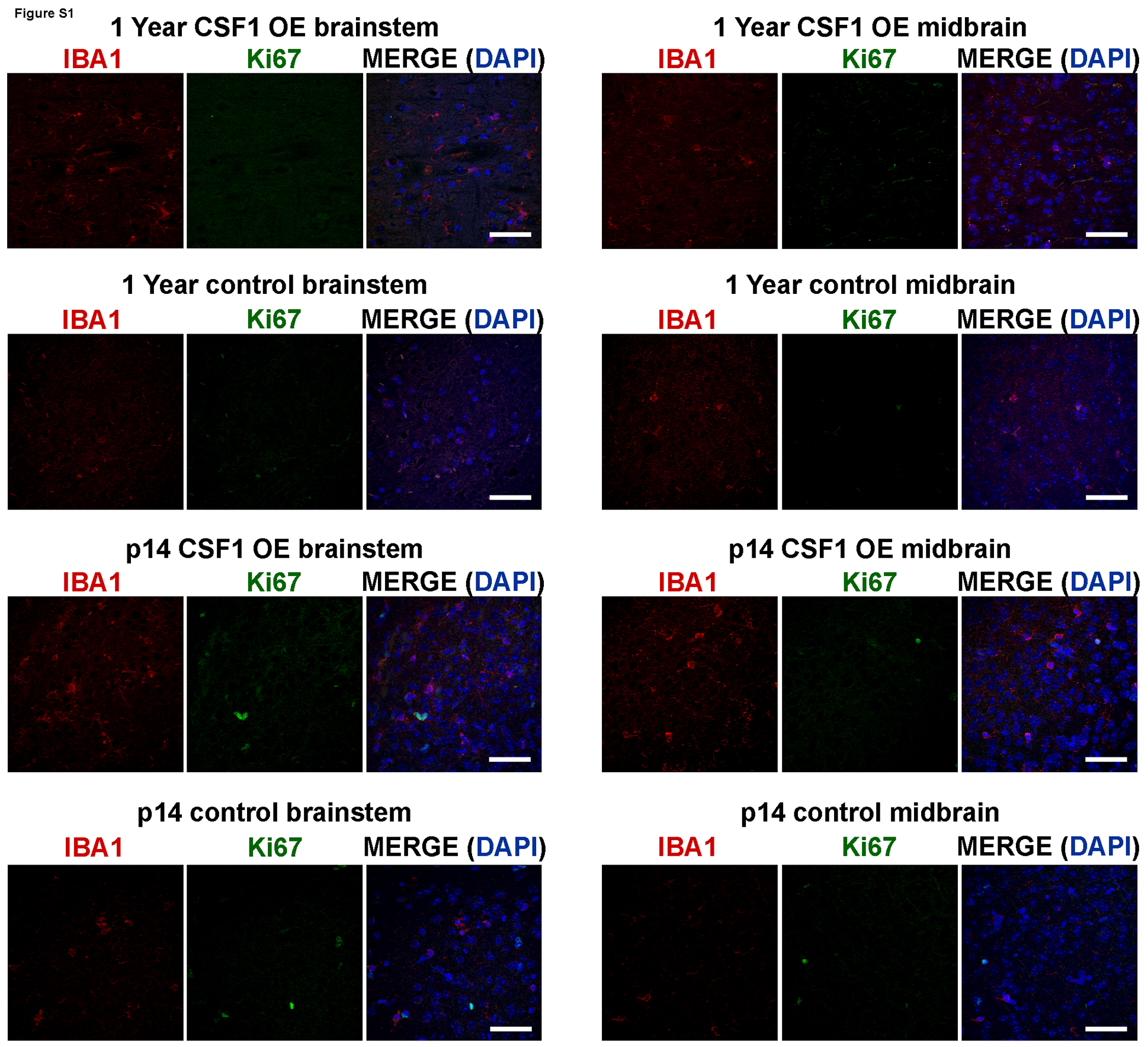

Supplement: Supplementary file 1 — Additional file 1: Supplemental Figure 1. Representative images for IBA1 and Ki67 immunofluorescence. Genotype, age, and brain region are indicated for each image. Scale bar = 50 microns. [file 12974_2021_2212_MOESM1_ESM.tif]

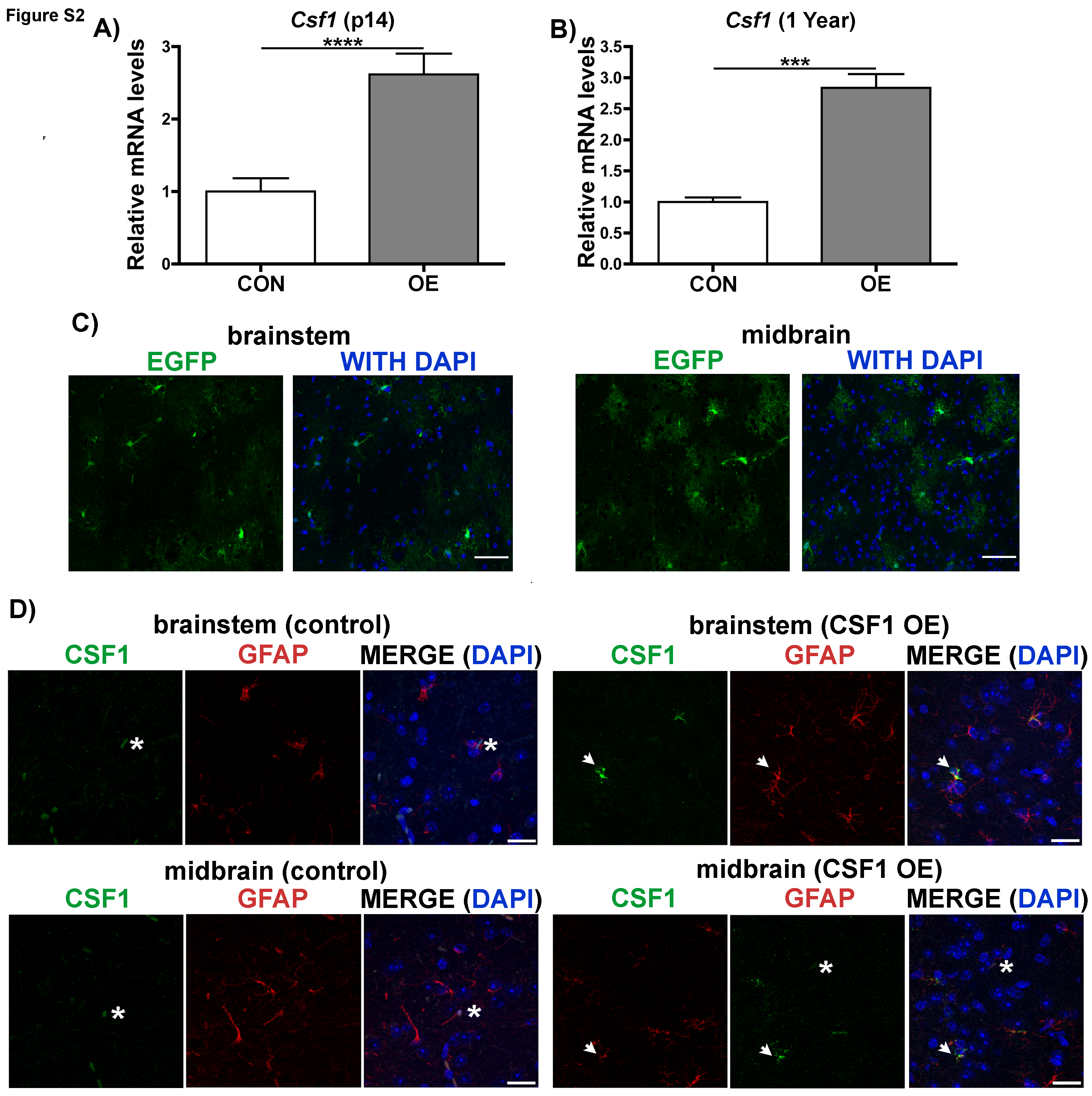

Supplement: Supplementary file 2 — Additional file 2: Supplemental Figure 2. Transgene expression in CSF1 OE mice. RT-qPCR indicates increased Csf1 levels in CSF1 OE (grey bars) mice compared to control mice (white bars) at both p14 (A) and 1 year (B). No-RT reactions were included for all samples and no amplification was detected (not shown). N=3-5 mice per group. ***= p<0.001; ****= p<0.0001; unpaired, two-tailed t-test. (C) Representative images showing expression of EGFP (green) in 1 year CSF1 OE mice. Scale bar = 50 microns. (D) Representative images showing detection of CSF1 protein by immunofluorescence in a subset of GFAP+ cells in 1-year old CSF1 OE mice but not control mice. Asterisks= examples of autofluorescence of red blood cells; arrows= examples of CSF1+ GFAP+ cells. Scale bar = 20 microns. [file 12974_2021_2212_MOESM2_ESM.tif]

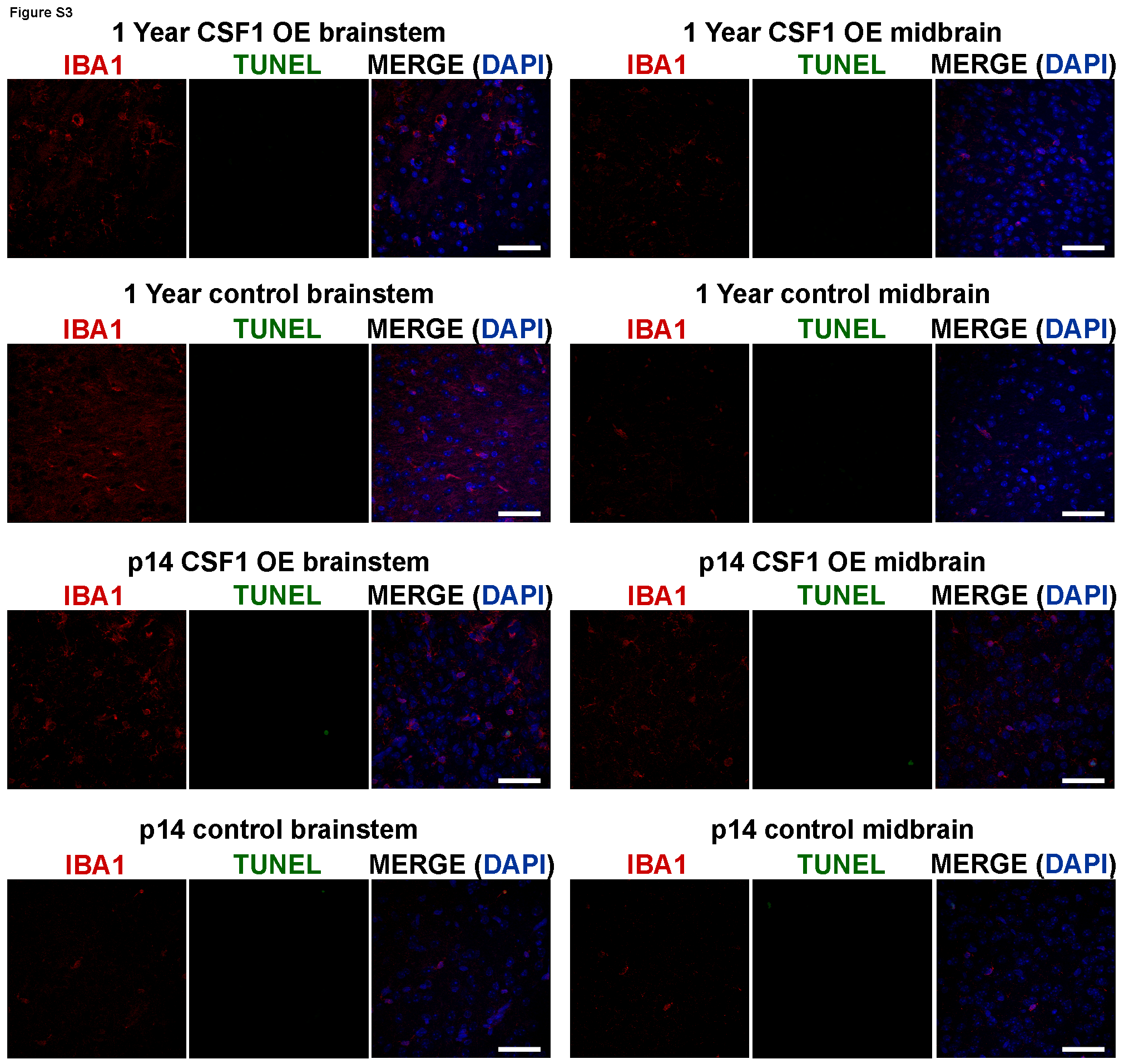

Supplement: Supplementary file 3 — Additional file 3: Supplemental Figure 3. Representative images for IBA1 and TUNEL immunofluorescence. Genotype, age, and brain region are indicated for each image. Scale bar = 50 microns. [file 12974_2021_2212_MOESM3_ESM.tif]

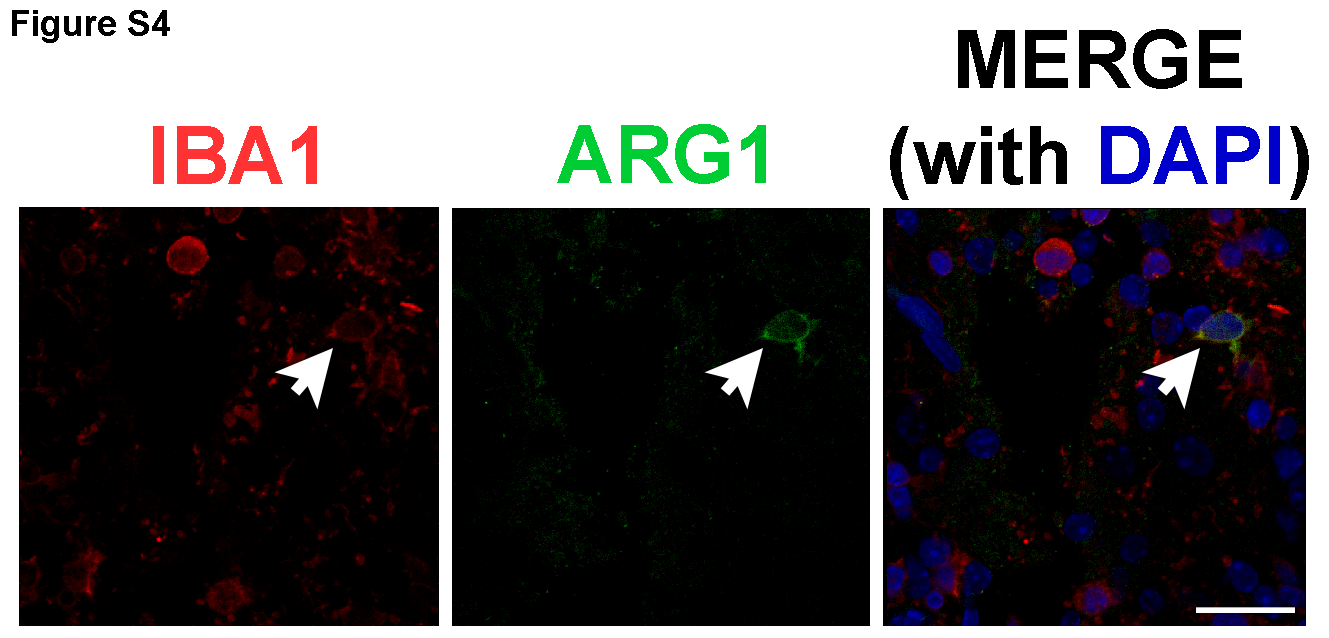

Supplement: Supplementary file 4 — Additional file 4: Supplemental Figure 4. ARG1 antibody validation. ARG1 (green) and IBA1 (red) immunofluorescence staining in a murine glioma. Arrow indicates an example ARG1+ IBA1+ cell. Scale bar = 25 microns. [file 12974_2021_2212_MOESM4_ESM.tif]

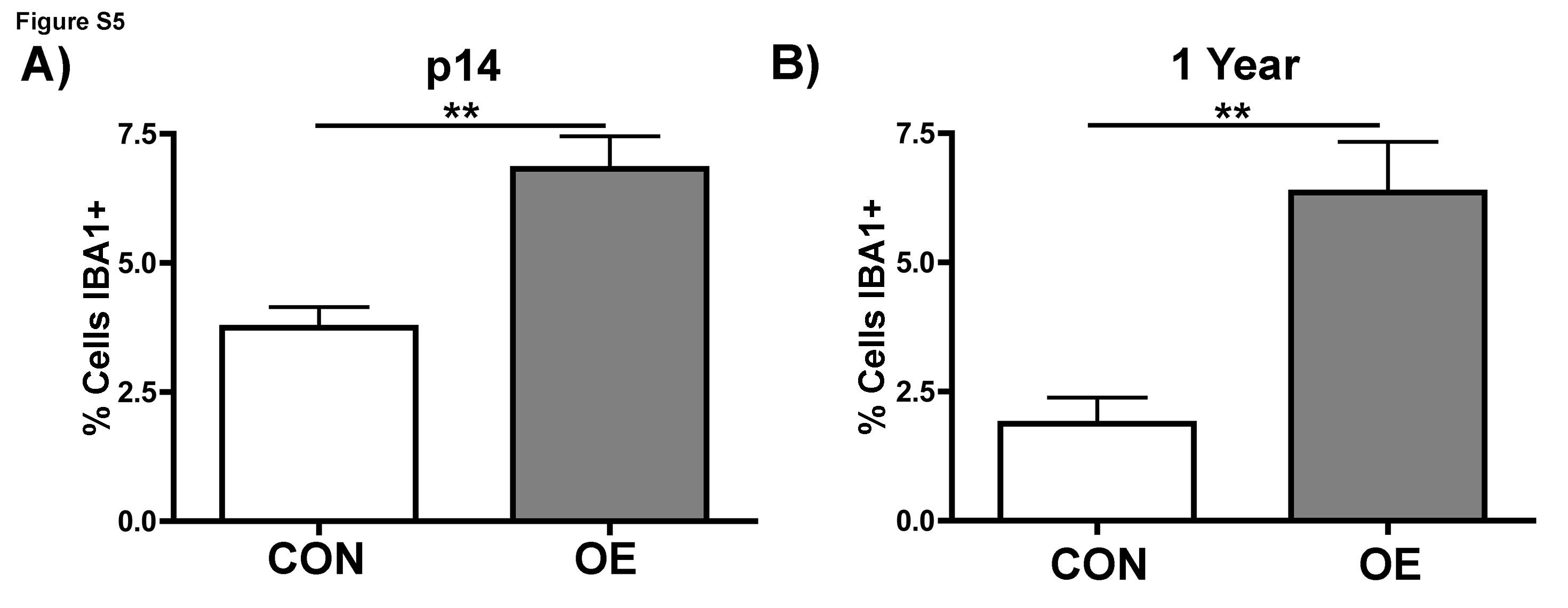

Supplement: Supplementary file 5 — Additional file 5: Supplemental Figure 5. IBA1+ cell numbers are increased in the cerebellar white matter of CSF1 OE mice. Quantification of the percent of cells that are IBA1+ in (CON, white bars) and CSF1 OE (OE, grey bars) mice at p14 (A) and 1 year (B). **=p<0.01 [file 12974_2021_2212_MOESM5_ESM.tif]

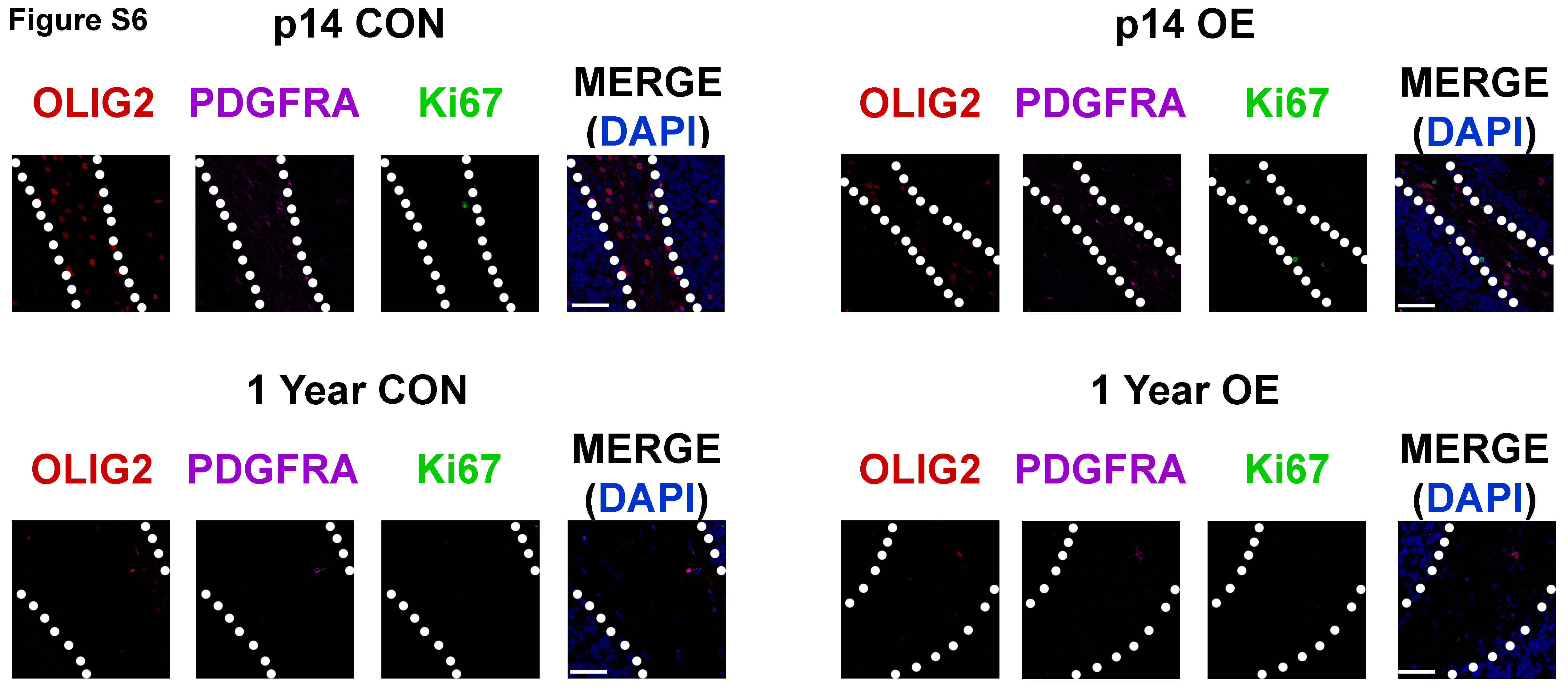

Supplement: Supplementary file 6 — Additional file 6: Supplemental Figure 6. Representative images for OLIG2, PDGFRA, and Ki67 immunofluorescence. Genotype and age are indicated for each image while dots indicate the edge of cerebellar white matter. Scale bar = 50 microns. [file 12974_2021_2212_MOESM6_ESM.tif]

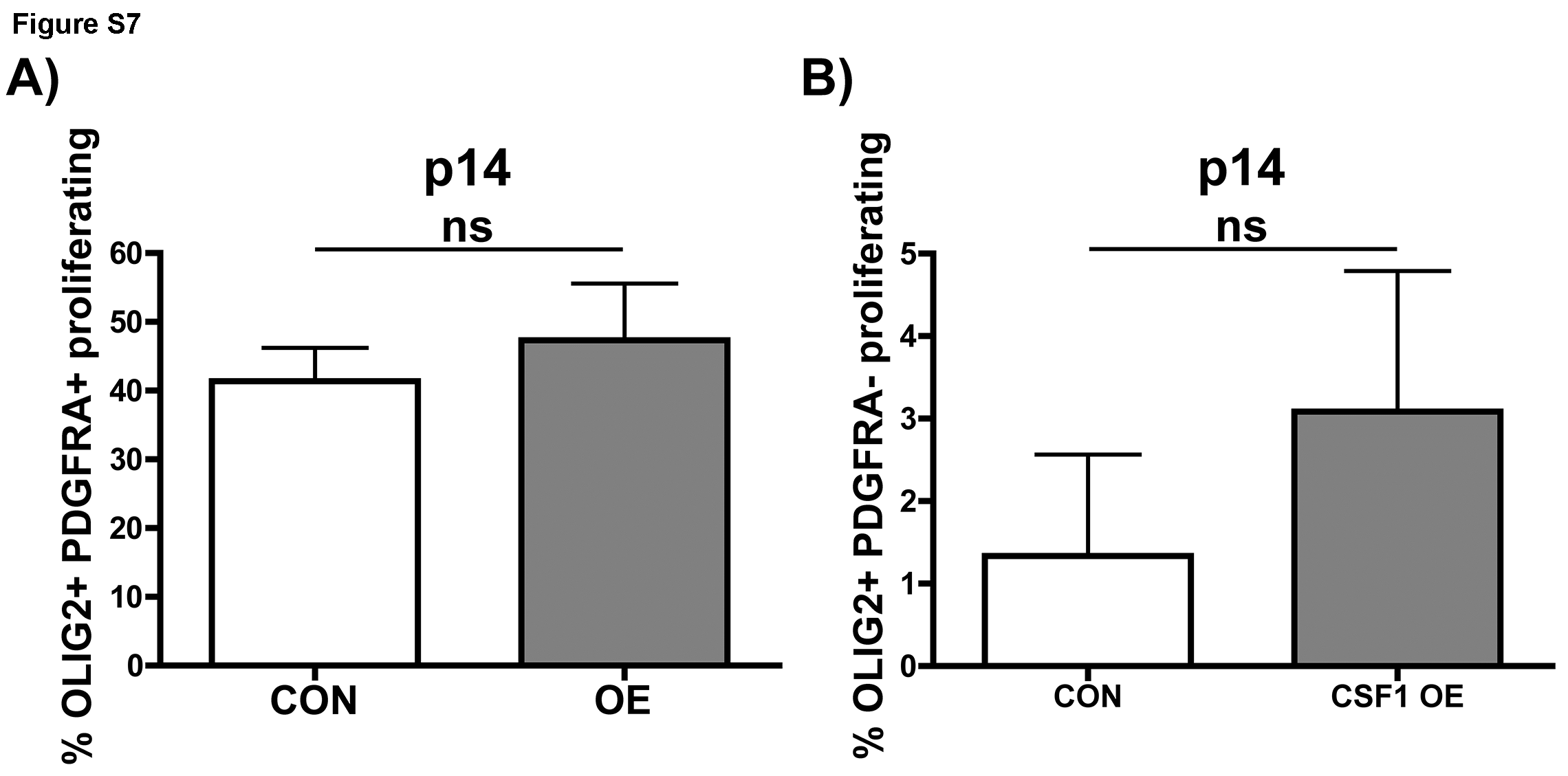

Supplement: Supplementary file 7 — Additional file 7: Supplemental Figure 7. Proliferation rates of oligodendrocyte lineage cells in the cerebellar white matter do not differ between control (CON, white bars) and CSF1 OE (OE, grey bars) mice. Quantification of the percent of OPCs (PDGFRA+; OLIG2+) (A) or mature or maturing oligodendrocytes (OLIG2+; PDGFRA-) (B) cells that are proliferating (Ki67+) at p14. No proliferating oligodendrocyte lineage cells were observed in 1-year old mice of either genotype. ns= non-significant (p>0.05), unpaired, two-tailed t-test. [file 12974_2021_2212_MOESM7_ESM.tif]

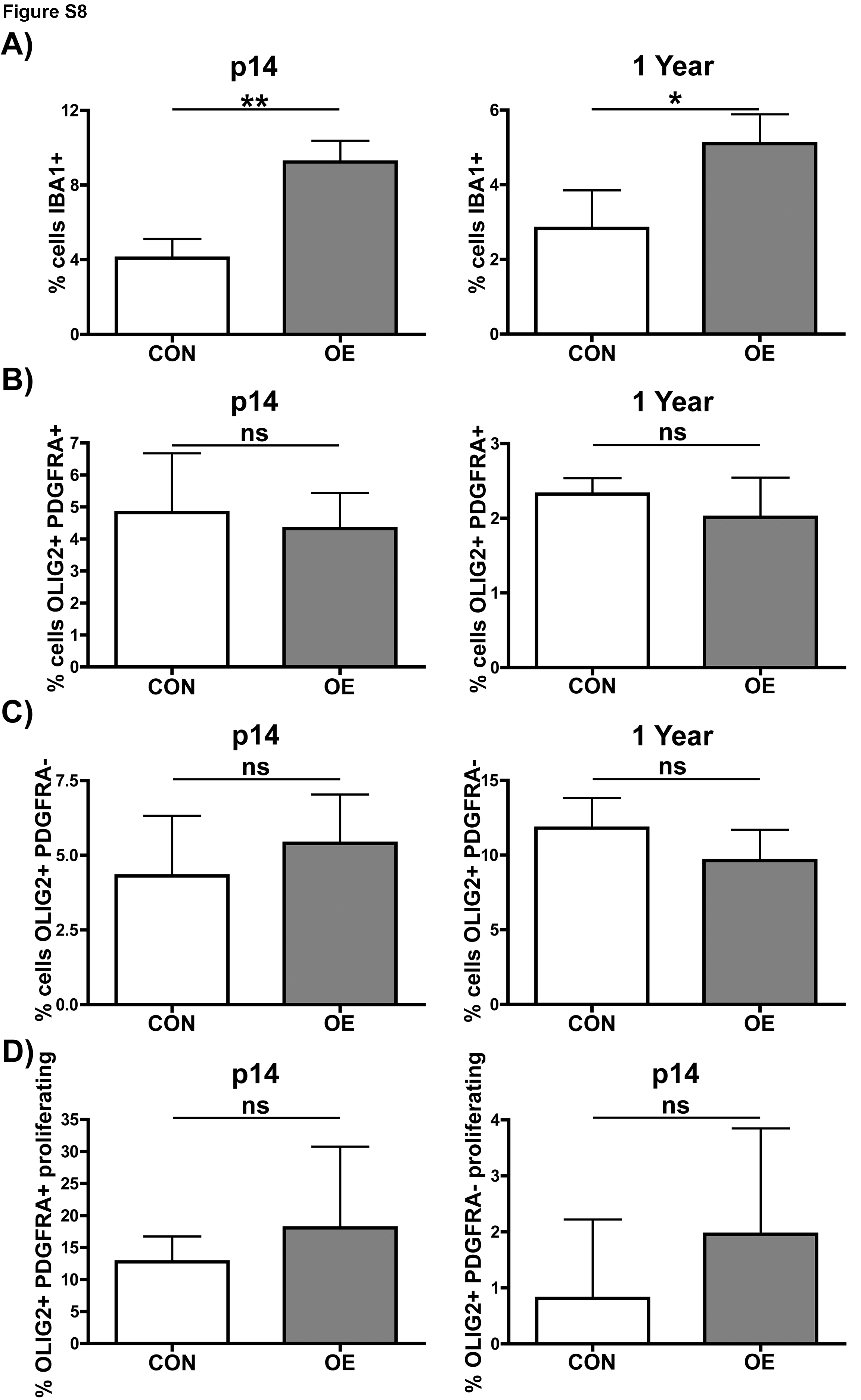

Supplement: Supplementary file 8 — Additional file 8: Supplemental Figure 8. IBA1+ cells are increased but there are no differences in oligodendrocyte lineage cells in the cortex of CSF1 OE mice. Quantification of the percent of cells that are IBA1+ (A), OLIG2+; PDGFRA+ (OPCs) (B), and mature or maturing oligodendrocytes (OLIG2+; PDGFRA-) (C) cells in control (CON, white shaded bars), and CSF1 OE (OE, grey shaded bars) mice at p14 and 1 year. D) Quantification of the percent of OPCs (PDGFRA+; OLIG2+) or mature or maturing oligodendrocytes (OLIG2+; PDGFRA-) cells that are proliferating (Ki67+) in control (CON, white shaded bars) and CSF1 OE (OE, grey shaded bars) mice at p14. No proliferating oligodendrocyte lineage cells were observed in 1-year old mice of either genotype. ns= non-significant (p>0.05), *=p<0.05, **=p<0.01, unpaired, two-tailed t-test. [file 12974_2021_2212_MOESM8_ESM.tif]

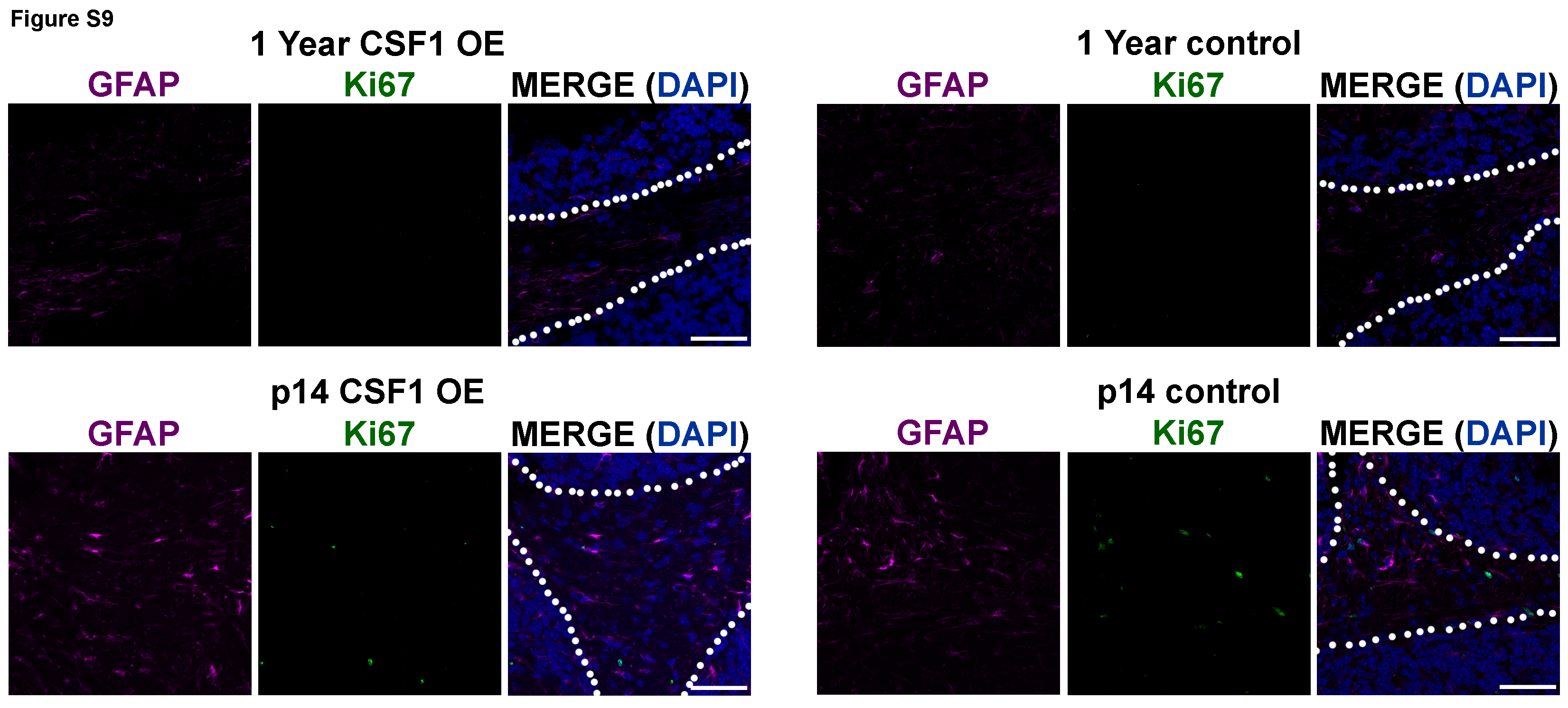

Supplement: Supplementary file 9 — Additional file 9: Supplemental Figure 9. Representative images for GFAP and Ki67 immunofluorescence. Genotype and age are indicated for each image while dots indicate the edge of cerebellar white matter. Scale bar = 50 microns. [file 12974_2021_2212_MOESM9_ESM.tif]
